# PKA-Dependent Membrane Surface Recruitment of CI-AMPARs Is Crucial for BCP-Mediated Protection Against Post-acute Ischemic Stroke Cognitive Impairment

**DOI:** 10.3389/fneur.2020.566067

**Published:** 2020-12-16

**Authors:** Sha Chen, Yuchun Wang, Xuhui Wang, Meng He, Lu Zhang, Zhi Dong

**Affiliations:** ^1^Key Laboratory of Biochemistry and Molecular Pharmacology, College of Pharmacology, Chongqing Medical University, Chongqing, China; ^2^Laboratory Sciences, Department of Clinical Biochemistry, Third Military Medical University (Army Medical University), Chongqing, China; ^3^Department of Neurosurgery, Research Institute of Surgery Daping Hospital, Third Military Medical University (Army Medical University), Chongqing, China; ^4^Chongqing Key Laboratory of Translational Medical Research in Cognitive Development and Learning and Memory Disorders, Children's Hospital of Chongqing Medical University, Chongqing, China

**Keywords:** GluA2-containing Ca^2+^ -permeable AMPA receptors, β-caryophyllene, post-acute ischemic stroke, synaptic plasticity, long-term potentiation, long-term depression, middle cerebral artery occlusion (MCAO) model, cAMP/PKA pathway

## Abstract

Post-acute ischemic stroke cognitive impairment frequently occurs and seriously affects patients daily activities. Recruitment of GluA2-containing Ca^2+^-impermeable AMPA receptors (CI-AMPARs) to hippocampal synaptic membrane surfaces was shown to trigger synaptic plasticity. Currently, the effect of CI-AMPAR trafficking on acute ischemic stroke remains poorly understood. β-Caryophyllene (BCP) has been shown to ameliorate cognitive impairment. However, the mechanism has not been characterized. In this study, a 60-min temporary middle cerebral artery occlusion (MCAO) model was established to simulate the pathology of acute ischemic stroke. BCP reduced neurologic deficits, cerebral infarct volume, and pathological damage in MCAO mice and caused CI-AMPARs to translocate to synaptic membranes in the hippocampus; surface expression of CI-AMPARs was also decreased in MCAO mice. Furthermore, this study also showed that BCP treatment significantly activated the cAMP/PKA pathway, which is consistent with the synaptic membrane expression of CI-AMPARs. To better understand the underlying mechanisms, the PKA inhibitor H-89 was used to study the role of BCP in MCAO mice. Interestingly, H-89 treatment significantly disrupted the BCP-mediated facilitation of CI-AMPAR translocation to the synaptic membrane surface and substantially attenuated BCP-induced protection against acute ischemic stroke. Additionally, inhibition the cAMP/PKA pathway not only reduced BCP-induced inhibition of AMPAR-mediated excitatory postsynaptic currents in the hippocampal CA1 region but also decreased the effect of BCP-mediated protection against post-acute ischemic stroke cognitive impairment. Taken together, these data indicate that PKA-dependent synaptic membrane surface recruitment of CI-AMPARs is crucial for the neuroprotective effect of BCP against acute ischemic stroke and protection against post-acute ischemic stroke cognitive impairment.

## Introduction

Cognitive impairment occurs frequently after acute ischemic stroke and leads to poor long-term outcomes (1). Prevention and treatment of cognitive impairment after acute ischemic stroke are essential for clinicians.

Alpha-amino-3-hydroxy-5-methyl-4-isoxazole propionic acid (AMPA) receptors (AMPARs) are the principle postsynaptic ionotropic glutamate receptors that mediate fast excitatory synaptic transmission in the central nervous system (CNS) (2, 3). AMPARs are composed of four types of subunits, designated as GluA1, GluA2, GluA3, and GluA4, which combine to form tetramers (4). GluA2 determines many of the major biophysical properties of AMPARs, including Ca^2+^ permeability, as Ca^2+^ plays a crucial role in many forms of synaptic plasticity (5). Thus, the GluA2 strongly influences AMPAR assembly and trafficking, and plays pivotal roles in a number of forms of long-term synaptic plasticity (6).

AMPARs can be classified based on whether they contain the GluA2 subunit, which decreases channel conductance and inhibits Ca^2+^ influx. GluA2-containing receptors are Ca^2+^-impermeable AMPARs (CI-AMPARs), while those lacking GluA2 are higher-conductance, Ca^2+^-permeable AMPARs (CP-AMPARs) (7, 8). The translocation of CI-AMPARs to the membrane surfaces of hippocampal synapses from extrasynaptic and/or intracellular locations contributes to the facilitation of LTP and LTD and triggering of synaptic plasticity (9, 10). LTP, a cellular model for long-term memory consolidation, involves early increases in CP-AMPARs, which are gradually replaced by CI-AMPARs (11). CI-AMPARs appear to be crucial mediators of AMPAR removal from the surface of the synaptic membrane in LTD (12–14).

Immediately after initiation of LTD, rapid endocytosis of extrasynaptic CI-AMPARs has been observed, followed after a delay by removal of AMPARs from the postsynaptic membrane (15).

Cannabinoid receptors are a group of G-protein coupled receptors that mediate retrograde synaptic signaling (16). Cannabinoid receptors include the cannabinoid receptor type 1 (CB1R) and cannabinoid receptor type 2 (CB2R) (17), and a CB2R agonist has shown neuroprotective effects against experimental ischemic stroke (18).

β-caryophyllene (BCP) is a bicyclic sesquiterpene compound, a full agonist of the CB2R (19, 20). Some studies have revealed that BCP exerts a prominent protective effect on cerebral neurons (21). Our group previously found that BCP pretreatment played a neuroprotective role in ischemia-reperfusion injury in SD rats (22, 23). Moreover, BCP has also been shown to ameliorate cognitive impairment and memory deficits in SD rats caused by chronic cerebral ischemia through the PI3K/Akt pathway (24). Additionally, BCP can effectively prevent AD via the PPARγ pathway (25). However, little is known about the role of BCP in post-acute ischemic stroke cognitive impairment from the perspective of synaptic plasticity.

The cyclic adenosine monophosphate (cAMP)/protein kinase A (PKA) pathway is important for learning and memory abilities (26). Recently, many studies have found that activation of the CB2R is associated with changes in the cAMP/PKA pathway (27), and CB2R activation ameliorates ischemic injury, potentially through modulation of cAMP/PKA signaling (28). Interestingly, specific CB2R agonists significantly suppress Ca^2+^ influx, which is mediated by the cAMP/PKA pathway (29). In addition, some studies have demonstrated that CI-AMPARs undergo constitutive insertion that is accelerated by PKA signaling (30). PKA phosphorylation also mediates scaling up of CI-AMPARs in hippocampal neurons (31). Cyclic adenosine monophosphate response element-binding protein (CREB) acts as a PKA downstream and plays important roles in neuronal survival, neurogenesis, and long-lasting activity-dependent synaptic plasticity (32). In mature neurons, BDNF as a CREB downstream plays a key role at synapses to promote memory formation (33). Currently, the effects of the cAMP/PKA pathway on BCP-mediated protection against post-acute ischemic stroke cognitive impairment are still unclear.

Our study aimed to determine whether PKA-dependent synaptic membrane surface recruitment of CI-AMPARs is crucial for the neuroprotective effect of BCP against acute ischemic stroke and for protection against post-acute ischemic stroke cognitive impairment.

## Materials and Methods

### Animals

Adult male C57BL/6 (20–25 g) mice in individual ventilated cages were obtained from the Experimental Animal Center, Chongqing Medical University (Chongqing, China). Water and food were available *ad libitum*. All mice were randomly divided into four groups (*n* = 6 in each group), including the sham-operated group (sham group), middle cerebral artery occlusion group (MCAO group), BCP-pretreated MCAO group (MCAO+BCP, dose = 72 mg/kg), and H-89 pretreated MCAO+BCP group (dose = 10 mg/kg).

### Middle Cerebral Artery Occlusion Model (MCAO)

Mice were anesthetized with 4% pentobarbital sodium (40 mg/kg, intraperitoneally). The mice were fixed in a supine position, a midline incision was made in the neck, and the right carotid artery (RCA), external carotid artery (ECA), and internal carotid artery (ICA) were separated from the right lateral sternocleidomastoid muscle and the anterior cervical muscle. The vagus nerve was separated into four regions at the ICA and ECA bifurcations. Then, RCA ligation was performed, and the ECA and the proximal ICA were blocked with a suspension line using the Zea-Longa method (34). Next, a 5 mm incision was made at the RCA bifurcation, and a kind of silicon nylon suture that was ~18 mm in length was inserted into the ICA. The filament was withdrawn gently after 60 min of occlusion, and the brain was then reperfused. The silicon nylon suture was not inserted in the sham-operated animals. Mice were returned to their cages for recovery and had free access to tap water and food. Throughout the procedure, body temperature was maintained at 37 ± 0.5°C with a thermostatically controlled infrared lamp.

### Drug Preparation and Treatment Procedures

BCP (Adamas, Switzerland) was dissolved in normal saline and 10% polyoxyethylated castor oil (EL) to prepare a suspension with a concentration of 72 mg/kg. The doses of BCP were chosen based on a previous study performed by our research group. The suspensions and solvent were given orally according to body weight once a day from the 1st day to the 6th day at the same time. The MCAO procedure was conducted 1 h after the last intragastric administration on the 6th day. The PKA inhibitor H-89 (10 mg/kg) (35) was administered on the 4th day.

### Neurologic Deficits

On the 7th day after MCAO operating, neurologic deficits were assessed by researchers who were unaware of the mouse group assignments (*n* = 6). The assessment was performed according to the Longa 5-level, 4-point method (36). The criteria for scoring were as follows: Grade 0: no observable deficit; Grade 1: mild forelimb weakness; Grade 2: severe forelimb weakness and consistent rotation to the side of the deficit when lifted by the tail; Grade 3: spontaneous circling or walking toward the contralateral side; Grade 4: walking only when stimulated or depressed level of consciousness.

### Rota-Rod Test

Rota-rod test was evaluated using a rota-rod (Ugo Basile, Italy). All groups of mice were placed on an accelerating rotarod cylinder. The rotating speed was increased from 4 to 40 rpm over 5 min. Rota-rod cylinder for 3 days prior to MCAO in order to obtain baseline measurements. The time for which all groups of the mice stayed on the rotarod was recorded by blinded researcher. The trial ended when the mice fell off the rungs or gripped the rod and spun around for two revolutions without attempting to walk on the rod. The mice were trained to perform this protocol three times per day for 3 consecutive days before MCAO operating, and the final average score on the last day before the surgery was considered the baseline. On days 1, 3, 5, and 7 post-MCAO, all groups of mice were placed on the rota-rod apparatus using the same acceleration conditions as above.

### Morris Water Maze Task

The MWM (ZS-001, ZSDichuang) consisted of a circular swimming tank with a diameter of 150 cm and depth of 60 cm. The tank was divided into four quadrants with different markers. A camera was installed above the tank to record the behavior of the mice. A 15-cm-wide circular platform was placed in the tank and submerged in opaque water. Each mouse was placed in the water in one of the four quadrants and allowed 60 s to find the platform. Mice that failed to locate the platform were guided to the platform and held there for 10 s. The MWM task was conducted in two stages. The first stage was the navigation test. On the 1st day, the platform was visible to the mice, and the time required to reach the platform was recorded. On subsequent days, the mice were tested twice a day, and the platform was hidden. The tests were continuously repeated for 5 days to examine the following indicators: escape latency, swimming track, number of times mice crossed the platform, and the time mice remained in the target quadrant. In the second stage, a spatial probe test was performed on the 7th day, and the platform was removed. The values of each indicator were recorded and analyzed to assess the learning and memory performances of the mice. The evaluator conducting the MWM tests was blinded to the experiment protocol.

### Evaluation of Infarct Volume

At 7 days after MCAO, TTC staining was performed. The brains of mice in each group were rapidly removed and cut into five 2-mm-thick slices. Coronal sections were stained with 2% TTC (Sigma-Aldrich, USA) for 30 min at 37°C in the dark and then fixed in 4% paraformaldehyde for 24 h at 4°C. The next day, the stained sections were photographed. Images were analyzed with Image J software. To correct for brain edema, the following formula was used: (contralateral volume – ipsilateral undamaged volume) × 100%/contralateral volume.

### Hematoxylin and Eosin Staining

At 7 days after MCAO, mice were deeply anesthetized, and intracardiac perfusion was performed with phosphate buffered saline (PBS) and 4% paraformaldehyde. The brain was quickly removed and fixed in 4% paraformaldehyde for 24 h. Ethanol in graded concentrations and xylene were then used to dehydrate the brain tissue, which was then embedded in paraffin. HE staining was performed on the paraffin sections (5 μm) according to the standard protocol. Histological analysis of the same region in each experiment was performed with a light microscope.

### Patch Clamp Technique

Mice were decapitated after being deeply anesthetized and perfused with ice-cold oxygenated (95% O_2_ and 5% CO_2_) dissection buffer consisting of the following (in mmol/L): 92 NMDG, 2.5 KCl, 1.25 NaH_2_${\text{PO}}_{4}^{*}$H_2_O, 30 NaHCO_3_, 20 HEPES, 25 glucose, 2 thiourea, 5 L-ascorbate, 3 sodium pyruvate, 12 NAC, 0.5 CaCl_2_, and 10 MgCl_2_, pH 7.4 (290–310 mOsm). Then, their brains were quickly removed. Coronal slices (350 μm) were cut in ice-cold oxygenated (95% O_2_ and 5% CO_2_) dissection buffer. Next, hippocampal slices were transferred to a recovery buffer containing the following (in mmol/L): 92 NaCl, 2.5 KCl, 1.25 NaH_2_${\text{PO}}_{4}^{*}$H_2_O, 30 NaHCO_3_, 20 HEPES, 25 glucose, 2 thiourea, 5 L-ascorbate, 3 sodium pyruvate, 12 NAC, 2 CaCl_2_, and 2 MgCl_2_, pH 7.4 (290–310 mOsm). The slices were bathed with artificial cerebrospinal fluid for 10 min at room temperature (25 ± 1°C). Slices were then transferred into artificial cerebrospinal fluid containing the following (in mmol/L): 119 NaCl, 2.5 KCl, 1.25 NaH_2_${\text{PO}}_{4}^{*}$H_2_O, 24 NaHCO_3_, 12.5 glucose, 2 CaCl_2_, and 2 MgCl_2_, pH 7.4 (290–310 mOsm). The slices were incubated for 1 h at 32°C. Slice recordings were performed in in artificial cerebrospinal fluid. Patch electrodes (4–6 MΩ resistance) were filled with an intracellular solution containing the following (in mmol/L): 122.5 Cs-gluconate, 17.5 CsCl, 0.2 EGTA, 10 HEPES, 1 MgCl_2_, 4 Mg-ATP, 0.3 Na-GTP, and 5 QX314, pH 7.2 (280–300 mOsm). Data were discarded if the series resistance changed by more than 20% during recording. A double EPC 10 (HEKA, Germany) amplifier was used under an BX51WIF microscope (Olympus, Japan) to record currents in CA1 pyramidal cells. AMPAR-mediated mEPSCs were recorded in the presence of tetrodotoxin (0.5 μmol/L), bicuculline (20 μmol/L), and APV (50 μmol/L). All recordings were performed at a holding potential of −70 mV, filtered at 2 kHz, and sampled at 10 kHz. Data were collected with Patchmaster software and analyzed with the Mini Analysis Program (Synaptosoft, Decatur, GA, USA) with an amplitude threshold of 5 pA for mEPSC analysis.

### Laser Confocal Immunofluorescence Staining

Mice were carefully anesthetized at 7 days after MCAO and then rapidly perfused with 0.9% sodium chloride. The brains was immediately removed and dehydrated with PBS containing 30% sucrose for 12 h. Afterwards, a frozen section slicer (Cryotome E, Thermo) was used to section the samples, which were then embedded and frozen in OCT-Freeze medium (4583, Sakura). Cryosections were incubated with 10% goat serum for 2 h at room temperature to block non-specific binding of immunoglobulin. Following blocking, the sections were incubated with rabbit anti-GluA2 antibodies (1:400, MAB397, Merck Millipore) at 4°C overnight as the primary antibody. After washing in PBS, the sections were incubated with the appropriate secondary antibody for 30 min at 37°C (DyLight 488 affiniPure goat anti-rabbit IgG, 1:100, A23220, Abbkine, USA). Synapses were determined via incubation with an antibody against synaptophysin (1:500, ab130436, Abcam). Subsequently, the slices were incubated with the secondary antibody (Alexa-594, A-11032) for 1 h. Nuclei were stained with 4′,6-diamidino-2-phenylindole (DAPI, C1006, Beyotime, China). Images were captured with a fluorescence microscope (Eclipse Ti-S, Nikon, Japan), and visual fields in each section were analyzed using Image-pro plus (IPP) 6.0 software.

### Cyclic Adenosine Monophosphate Assay

The cAMP levels in the hippocampus were measured using an ELISA kit (Elabscience Biotechnology Co., Ltd, China). At 7 days after MCAO, mouse hippocampus tissues were homogenized in PBS, and then the homogenates were centrifuged at 5,000 × g for 15 min at 4°C to remove the particles. The cAMP levels are expressed as pmol/mg protein.

### Biotinylation Assays

Properly anesthetized mice were decapitated to obtain the right-side brain tissues at 7 days after MCAO, after which, tissues were rapidly extracted on ice, weighed, and ground in liquid nitrogen. Then, ~20 mg of homogenate was weighed. The samples were resuspended in 50 μL of PBS and then incubated in 200 μL of a solution containing 1.5 mg·mL^−1^ of a sulfo-NHSSS-biotin moiety (Pierce) at 4°C for 30 min to biotinylate surface proteins. Unreacted biotin was then quenched and removed with 100 mM glycine in PBS. After centrifugation (1,000 × g, 10 min, 4°C), the cell membranes were lysed in homogenization buffer containing 1% SDS and a protease and phosphatase inhibitor mixture (Roche). Biotinylated proteins were precipitated with 200 μL of Pierce avidin agarose for 2 h at 4°C. The agarose beads were precipitated by sequential centrifugation (500 × g, 3 min) followed by three washes with homogenization buffer. After the final precipitation, the washed beads were eluted twice with sample buffer and heated for 10 min at 98°C.

### Western Blot Assessment

Hippocampal samples from mice were prepared as described previously. The frozen tissue was transferred to a 1.5-mL centrifuge tube with RAPI lysis buffer (P00113D; Beyotime, Shanghai, China) that contained a proteinase and phosphatase inhibitor cocktail and centrifuged at 12,000 × g for 15 min at 4°C. The protein samples were harvested and lysed in RIPA buffer (Beyotime, China). The protein concentration was determined using a BCA protein assay kit (Beyotime, China). Then, the total proteins and surface proteins prepared by biotinylation assays (40 μg/well) were subjected to 10% sodium dodecyl sulfate-polyacrylamide gel electrophoresis. After electrophoresis, the proteins were transferred onto a polyvinylidene fluoride membrane (Bio-Rad, USA), blocked, and incubated with various primary antibodies at 4°C overnight. Antibodies against GluA2 (1:800, MAB397, Merck Millipore), PKA (1:1,000, #5842, Cell Signaling), p-PKA (1:1,000; sc-377575, Santa Cruz Biotechnology), p-CREB (1:1,000, #9198, Cell Signaling), CREB (1:1,000, #9197, Cell Signaling), BDNF (1:800, 28205-1-AP,Proteintech), and β-actin (1:10,000, 60008-1-Ig,Proteintech) were used as primary antibodies. The membranes were washed and incubated with secondary antibodies and then detected using an ECL kit (Millipore, USA).

### Statistical Analysis

Statistical analysis was performed by SPSS v17.0 software. All data are presented as the mean ± SD, and groups were compared with one-way analysis of variance followed by Tukey's test. A value of *p* < 0.05 was considered to indicate statistical significance.

## Results

### BCP Ameliorates Acute Ischemic Stroke-Induced Brain Injury in C57BL/6 Mice

**Figure 1:**
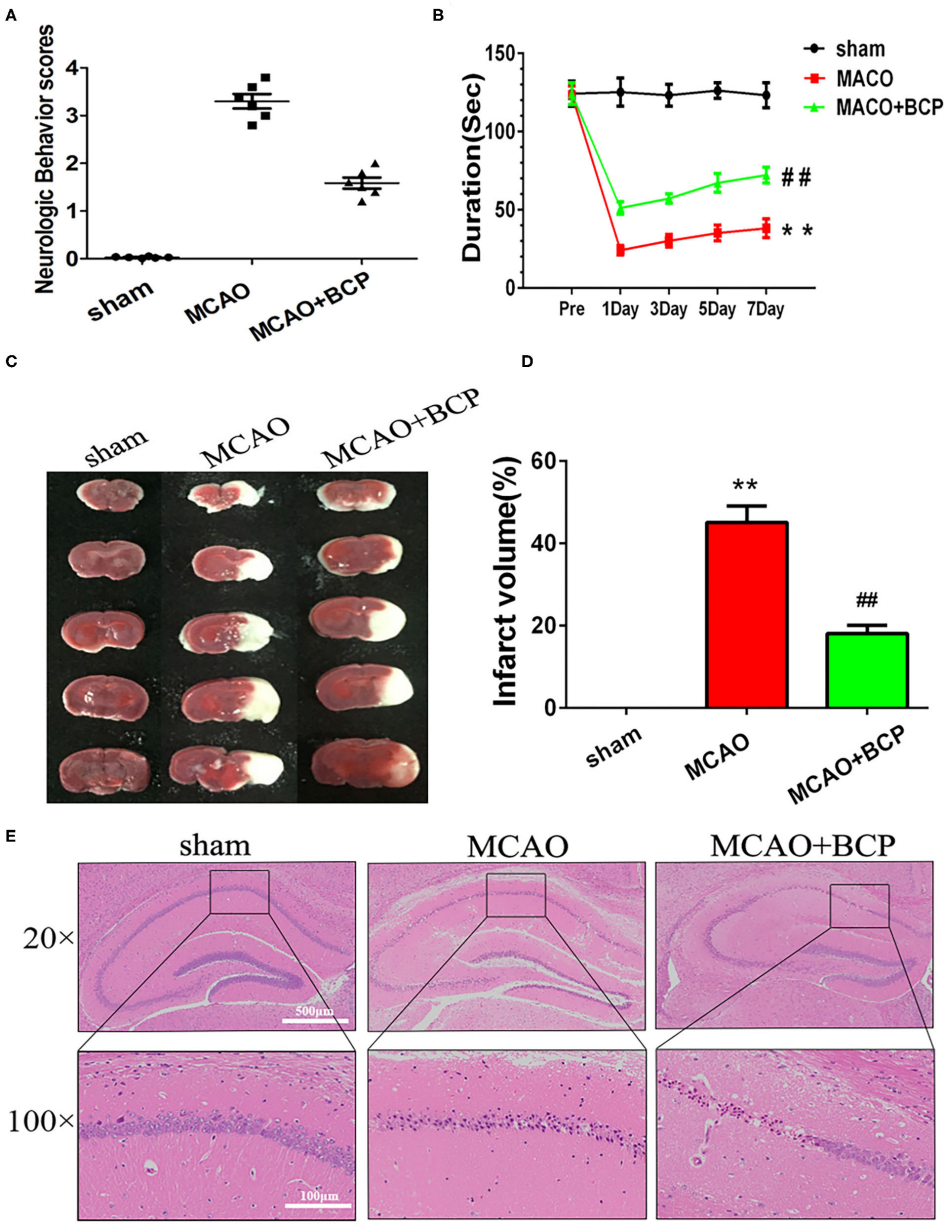
β-caryophyllene ameliorates acute ischemic stroke-induced brain injury in C57BL/6 mice**. (A)** Quantitative analysis of neurological function (*n* = 6 in each group). **(B)** The duration of the rotarod test of all groups mice **(***n* = 6 in each group). **(C)** 2, 3, 5-triphenyl-tetrazolium-chloride staining of brain slices (*n* = 6 in each group). **(D)** Quantitative analysis of the cerebral infarct volume. **(E)** Hematoxylin and eosin staining of brain slices (*n* = 6 in each group, 2 mm for each scale). **p < 0.01 vs. the sham group, ^*##*^*p* < 0.01 vs. the MCAO group.

To determine whether BCP contributes to protection against acute ischemic stroke, C57BL/6 mice underwent a middle cerebral artery occlusion (MCAO) procedure. As shown in Figure 1A, severe neurological deficits were present in the MCAO group, while BCP treatment (72 mg/kg) reduced the neurological score. MCAO group mice reduced the time on the rotating rod in comparison with the respective sham-operated groups, While, treatment with BCP increased the time spent on the rod at 1, 3, 5, and 7 days after MCAO operating (Figure 1B). Those suggests that BCP treatment improves the functional outcome after MCAO. Additionally, consecutive brain sections stained with 2, 3, 5-triphenyl-tetrazolium-chloride (TTC) were also examined. Compared with the MCAO group, BCP treatment (72 mg/kg) decreased the total infarct volume (Figures 1C,D, ^**^p < 0.01 vs. the sham group, ^*##*^*p* < 0.01 vs. the MCAO group). Hematoxylin and eosin (HE) staining showed that compared with the MCAO group, the BCP group exhibited less neuronal cell death in the hippocampal CA1 region, indicating that BCP plays a neuroprotective role (Figure 1E). The TTC and HE staining results indicated that BCP treatment decreased the ischemic area in the brain caused by acute ischemic stroke.

### BCP Facilitates CI-AMPAR Translocation to the Synaptic Membrane and Recruitment of These Receptors in Acute Ischemic Stroke

**Figure 2:**
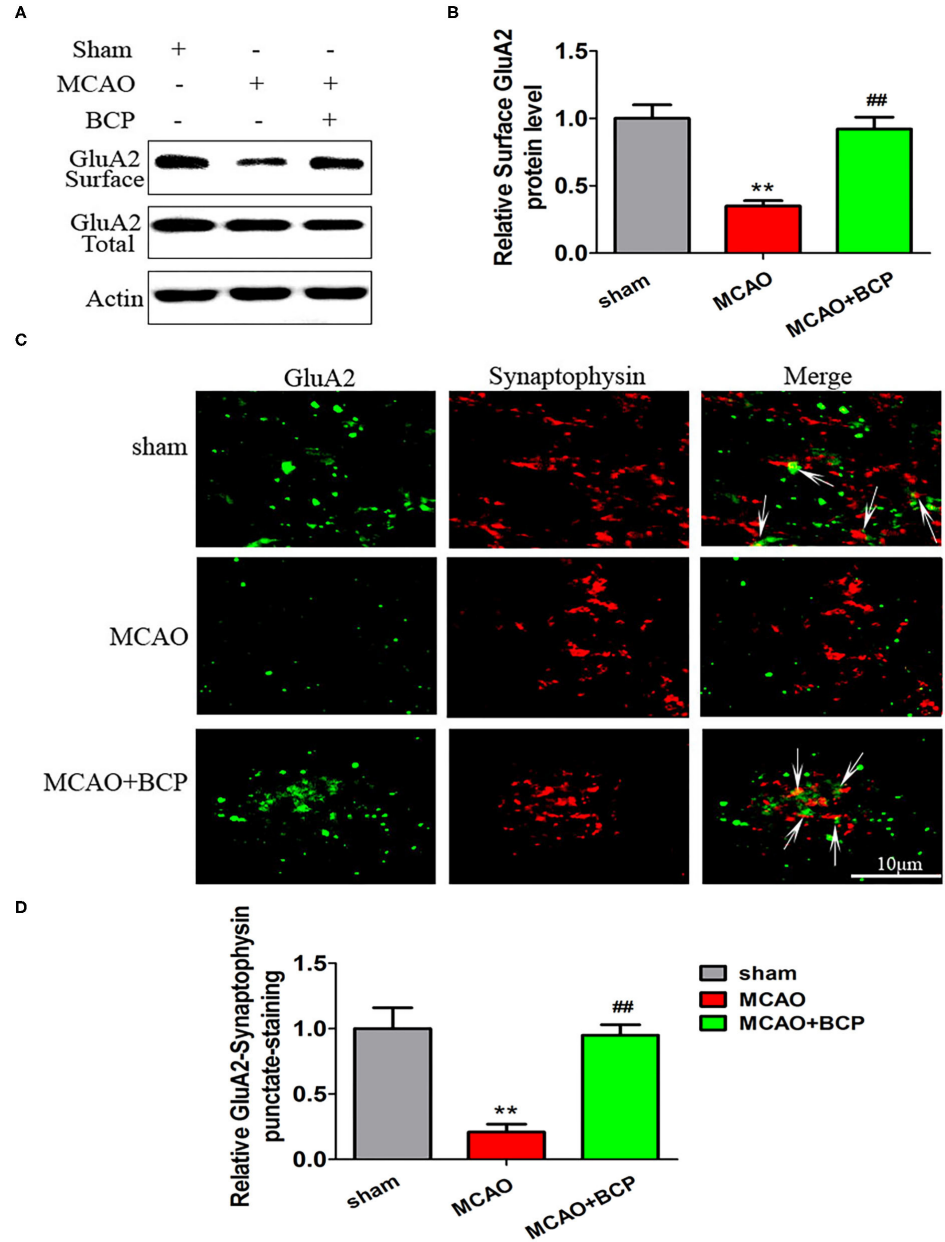
β-caryophyllene facilitates translocation of GluA2-containing Ca^2+^-impermeable AMPA receptors to the synaptic membrane and recruitment of these receptors in acute ischemic stroke. **(A,B)** Protein expression of the GluA2 AMPA receptor subunit on the surface of the synaptic membrane of mouse hippocampal cells was determined by western blotting and densitometric analysis using β-actin as an internal control (*n* = 6 in each group). **(C,D)** Images of hippocampal CA1 neurons double-labeled for the GluA2 AMPAR subunit (green) and synaptophysin (red) and quantitative data determined by laser confocal immunofluorescence staining. **p < 0.01 vs. the sham group, ^*##*^*p* < 0.01 vs. the MCAO group.

To determine the expression of CI-AMPARs on the synaptic membrane surface in the acute ischemic stroke model, western blots were used to detect the surface levels of the AMPAR subunit GluA2 and total GluA2 expression. The results showed that the MCAO group exhibited significantly reduced expression of the GluA2 subunit (Figures 2A,B ^**^p < 0.01 vs. the sham group, ^*##*^*p* < 0.01 vs. the MCAO group) on the synaptic membrane surface compared with the sham group. However, BCP treatment (72 mg/kg) increased expression of the GluA2 subunit on the synaptic membrane surface compared with the MCAO group. No significant difference in total GluA2 expression was found between in these two groups.

Additionally, images of CA1 neurons double-labeled for the GluA2 subunit (green) and synaptophysin (red) and quantitative data determined by laser confocal immunofluorescence analysis are illustrated in Figures 2C,D. The data showed that co-localization of GluA2 with synaptophysin in the MCAO group was significantly decreased compared with that in the sham group, whereas a marked increase was noted after treatment of MCAO mice with BCP compared with the MCAO group (^**^p < 0.01 vs. the sham group, ^*##*^*p* < 0.01 vs. the MCAO group). These results indicate that BCP treatment is essential for translocation of CI-AMPARs to the synaptic membrane and the recruitment of these receptors during acute ischemic stroke.

### BCP Activates the cAMP/PKA Pathway in Acute Ischemic Stroke

**Figure 3:**
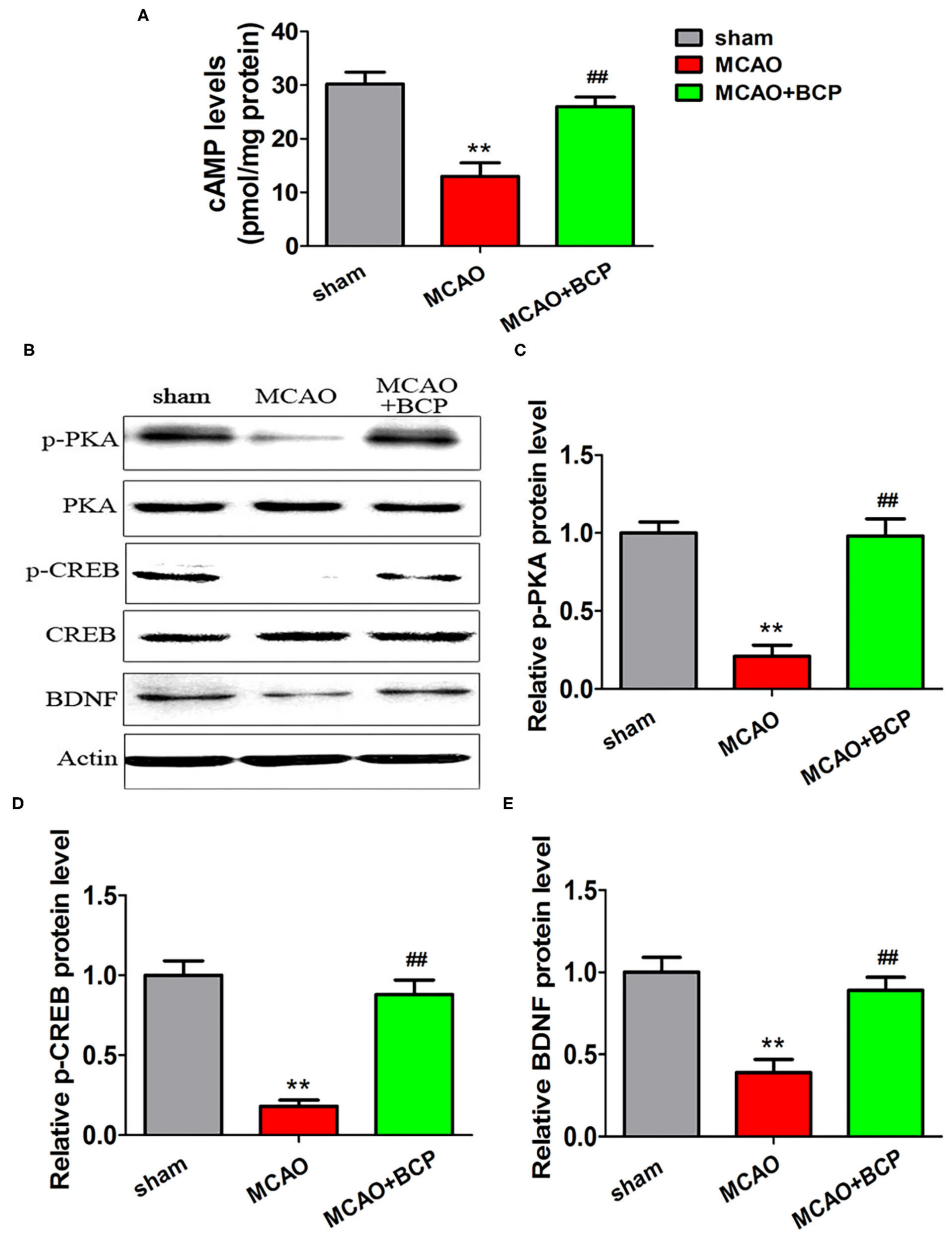
β-caryophyllene activates the cAMP/PKA pathway in acute ischemic stroke. **(A)** The expression of cAMP in the hippocampal cells of different groups of mice was examined by an enzyme-linked immunosorbent assay. **(B)** p-PKA, PKA, p-CREB, CREB, and BDNF expression levels in the hippocampus of mouse groups were determined at 7 days after middle cerebral artery occlusion by western blotting (*n* = 6 in each group). **(C)** p-PKA/PKA ratios. **(D)** p-CREB/CREB ratios. **(E)** BDNF/actin. **p < 0.01 vs. the sham group, ^*##*^*p* < 0.01 vs. the MCAO group.

To elucidate the effect of BCP on the cAMP/PKA signaling pathway in the acute ischemic stroke mouse model, we assessed the expression of cAMP with an enzyme-linked immunosorbent assay (ELISA) and the expression of phosphorylated PKA (p-PKA), PKA, phosphorylated CREB (p-CREB), CREB, and BDNF by western blotting. As depicted in Figures 3A–E, sharp downregulation of cAMP, p-PKA, p-CREB, and BDNF expression was observed in the ELISA and western blot assays in the MCAO group compared with those in the sham group. However, compared with the MCAO group, the BCP-treated group showed less degradation of cAMP, p-PKA, p-CREB, and BDNF expression (^**^p < 0.01 vs. the sham group, ^*##*^*p* < 0.01 vs. the MCAO group). These results indicated that BCP activated the cAMP/PKA pathway in the acute ischemic stroke mouse model.

### The cAMP/PKA Pathway Is Involved in BCP-Induced Neuroprotection in Acute Ischemic Stroke

**Figure 4:**
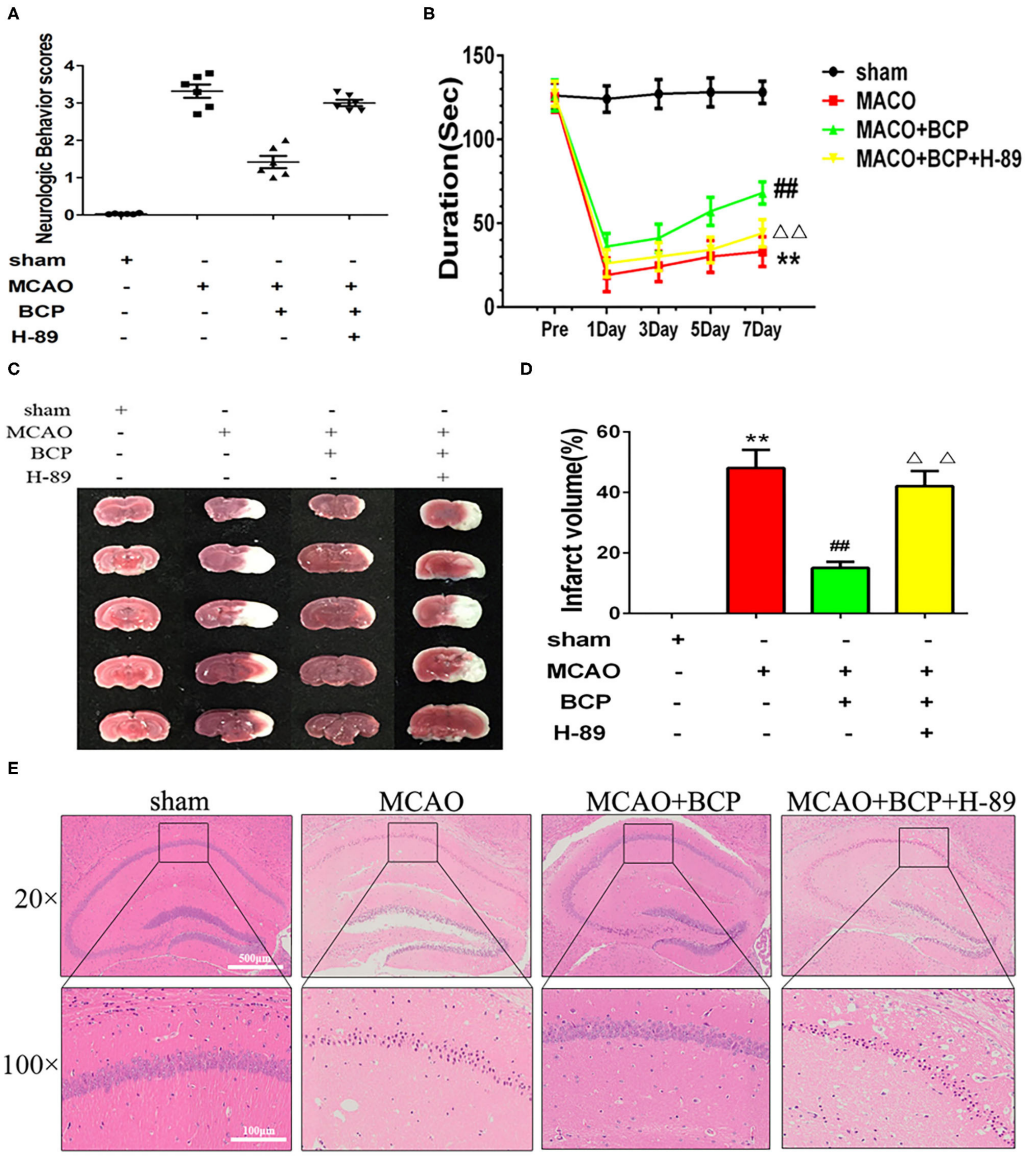
The cAMP/PKA pathway is involved in β-caryophyllene (BCP)-induced neuroprotection in acute ischemic stroke **(A)** Quantitative analysis of neurological function (*n* = 6 in each group). **(B)** The duration of the rotarod test of all groups mice **(***n* = 6 in each group). **(C)** 2, 3, 5-triphenyl-tetrazolium-chloride staining of brain slices (*n* = 6 in each group, 2 mm for each scale). **(D)** Quantitative analysis of cerebral infarct volume. **(E)** Hematoxylin and eosin staining of brain slices (*n* = 6 in each group, 2 mm for each scale). **p < 0.01 vs. sham the group, ^*##*^*p* < 0.01 vs. the MCAO group, ^ΔΔ^*p* < 0.01 vs. the BCP group.

**Figure 5:**
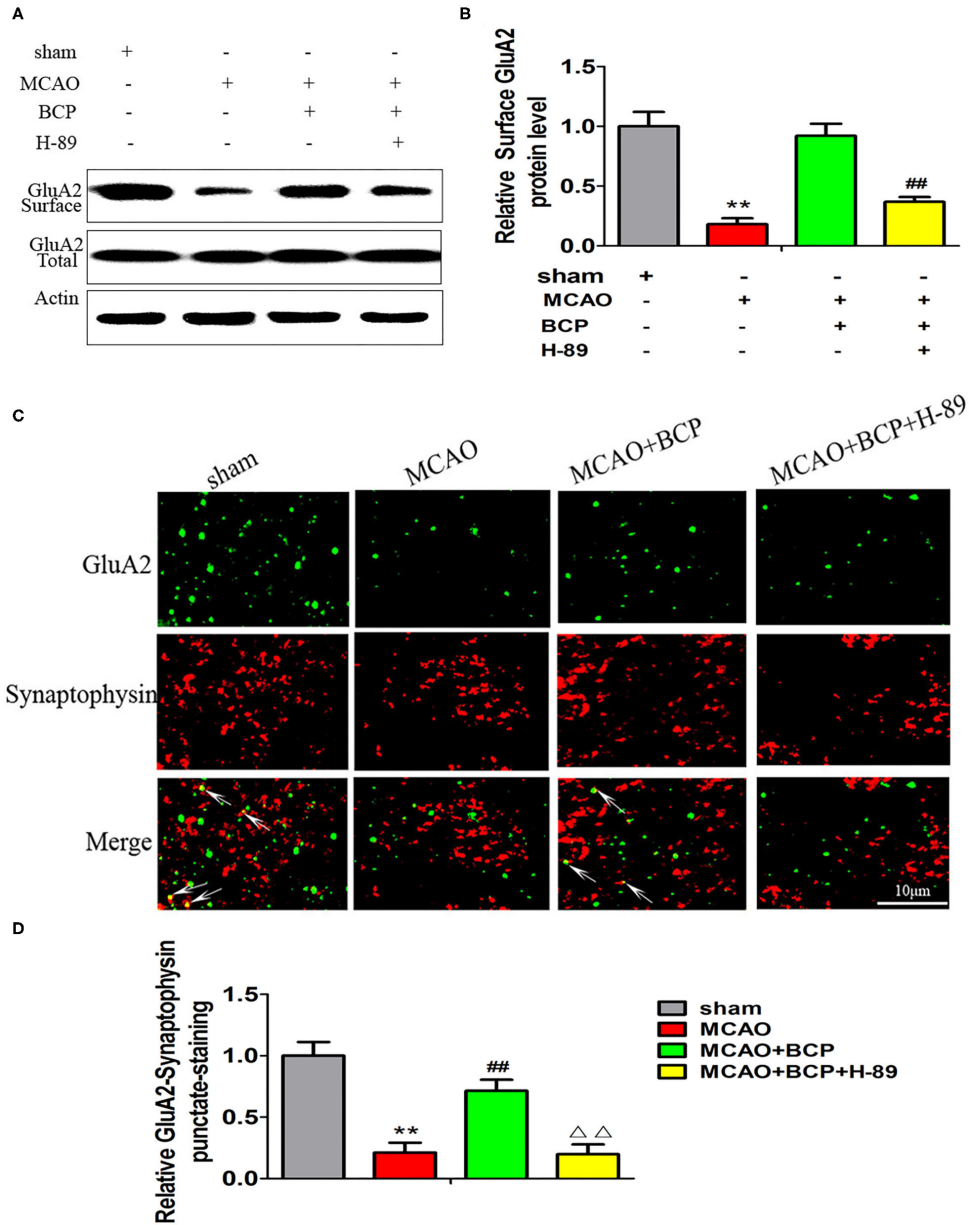
The cAMP/PKA pathway is involved in β-caryophyllene (BCP)-induced facilitation of GluA2-containing Ca^2+^-impermeable AMPA receptor translocation to the synaptic membrane and recruitment of these receptors in acute ischemic stroke **(A,B)** Protein expression of the GluA2 AMPA receptor subunit on the surface of the synaptic membrane of mouse hippocampal cells was determined by western blotting and densitometric analysis using β-actin as an internal control (*n* = 6 in each group). **(C,D)** Images of hippocampal CA1 neurons double-labeled for the GluA2 AMPA receptor subunit (green) and synaptophysin (red) with quantitative data detected by laser confocal immunofluorescence staining. **p < 0.01 vs. the sham group, ^*##*^*p* < 0.01 vs. the MCAO group, ^ΔΔ^*p* < 0.01 vs. the BCP group.

**Figure 6:**
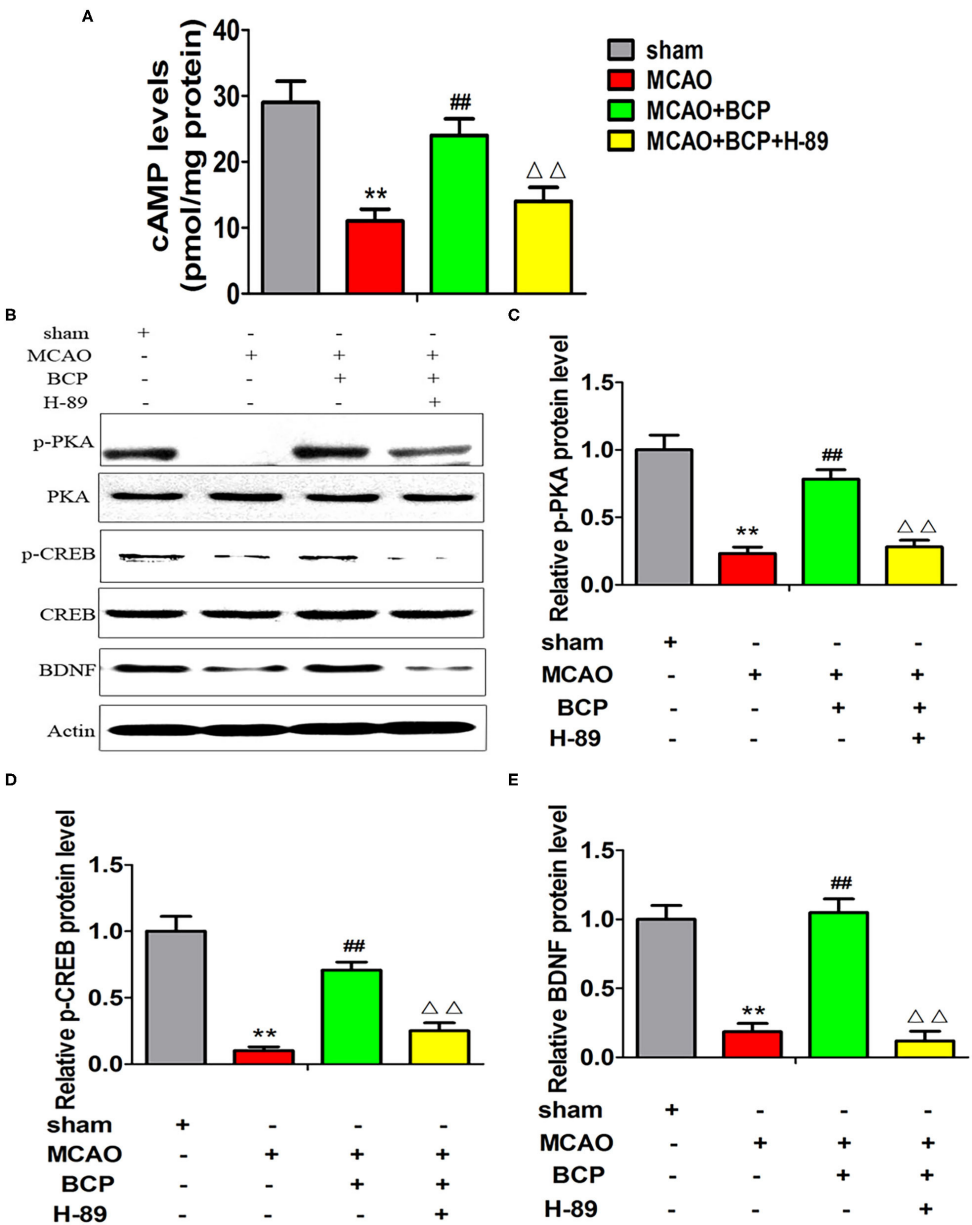
Expression of cAMP/PKA pathway-related proteins in each group after treatment with the inhibitor H-89. **(A)** The expression of cAMP in the hippocampus of different groups of mice was examined by enzyme-linked immunosorbent assays. **(B)** p-PKA, PKA, p-CREB, CREB, and BDNF expression levels in the hippocampus of different groups of mice were determined at 7 days after middle cerebral artery occlusion by western blotting (*n* = 6 in each group). **(C)** p-PKA/PKA ratios. **(D)** p-CREB/CREB ratios. **(E)** BDNF/actin ratios. **p < 0.01 vs. the sham group, ^*##*^*p* < 0.01 vs. the MCAO group, ^ΔΔ^*p* < 0.01 vs. the BCP+MCAO group.

**Figure 7:**
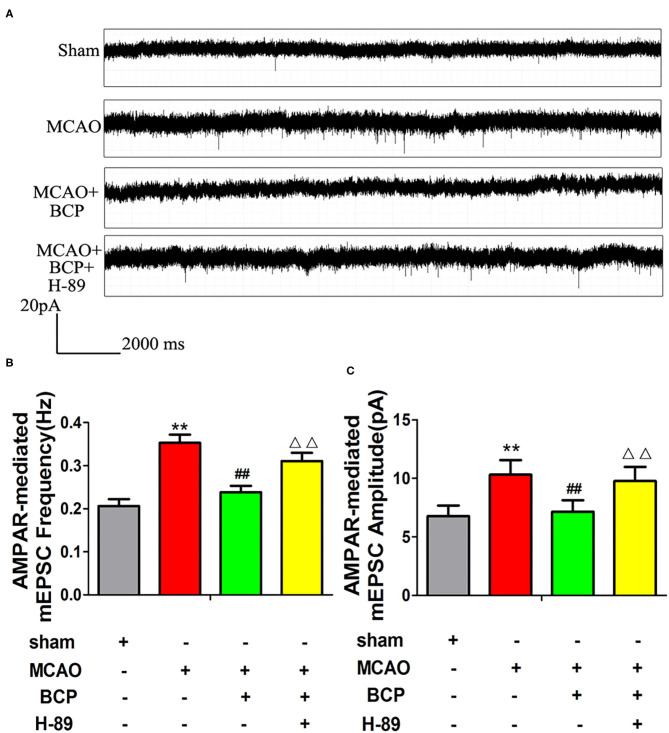
The cAMP/PKA pathway is involved in β-caryophyllene (BCP)-induced inhibition of AMPA receptor-mediated miniature excitatory post synaptic currents (mEPSCs). **(A)** Patch clamp recordings show representative mEPSCs mediated by AMPA receptors in the different groups. **(B,C)** Cumulative probabilities and average mEPSC amplitudes and frequencies from the different treatment groups (*n* = 6 in each group). **p < 0.01 vs. the sham group, ^*##*^*p* < 0.01 vs. the MCAO group, ^ΔΔ^*p* < 0.01 vs. the BCP+MCAO group.

**Figure 8:**
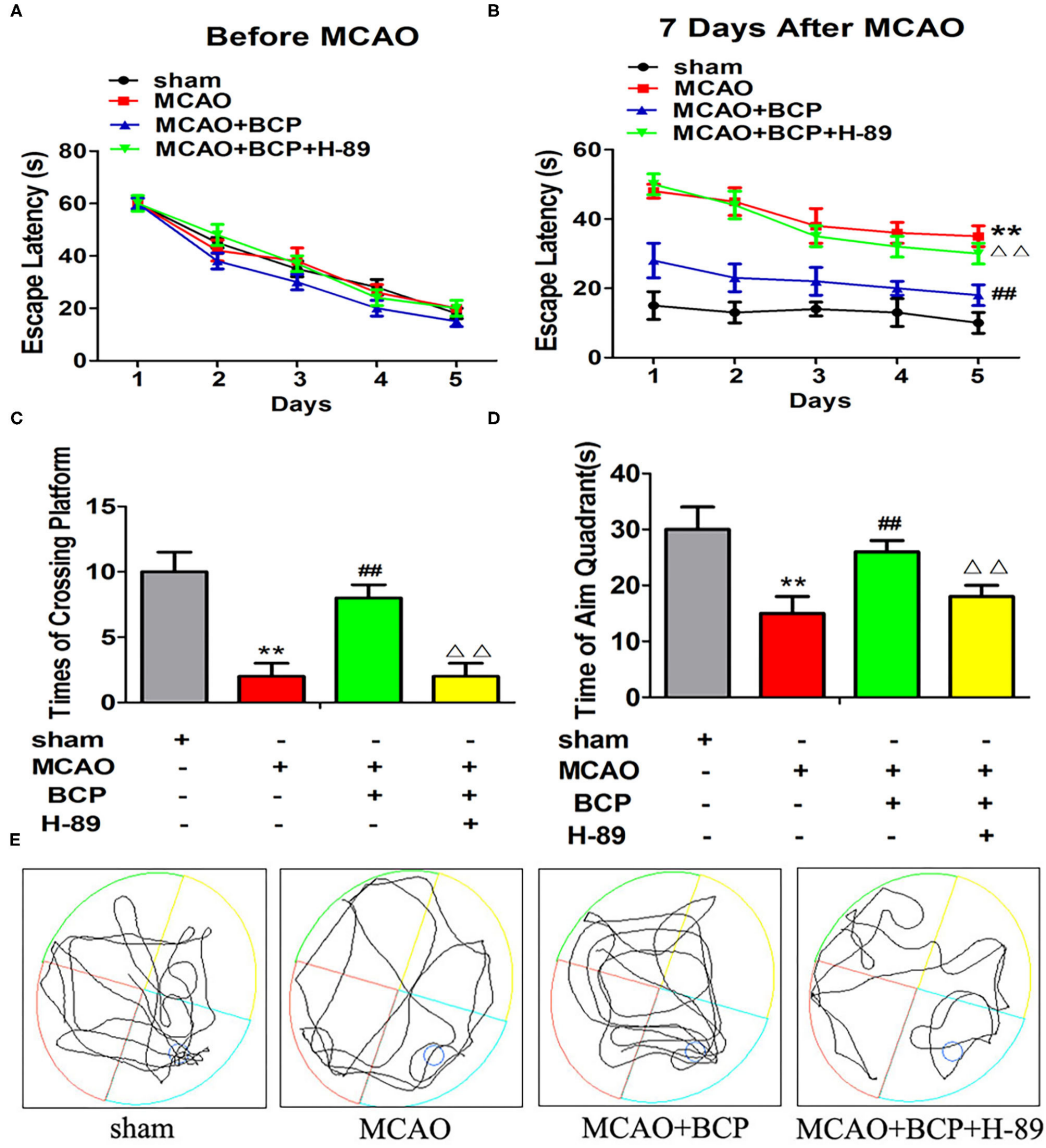
Facilitation of GluA2-containing Ca^2+^-impermeable AMPA receptor translocation to the synaptic membrane surface is dependent on the cAMP/PKA pathway, which is also required for β-caryophyllene (BCP)-mediated protection against post-acute ischemic stroke cognitive impairment. **(A)** The latency in locating the platform in different groups of mice before middle cerebral artery occlusion (MCAO). **(B)** The change in escape latency at 7 days after MCAO treatment. **(C)** Number of times that each group traversed the platform. **(D)** The time that each group remained in the target quadrant. **(E)** Trajectory of each group in the water maze (*n* = 6 in each group). **p < 0.01 vs. the sham group, ^*##*^*p* < 0.01 vs. the MCAO group, ^ΔΔ^*p* < 0.01 vs. the BCP+MCAO group.

**Figure 9:**
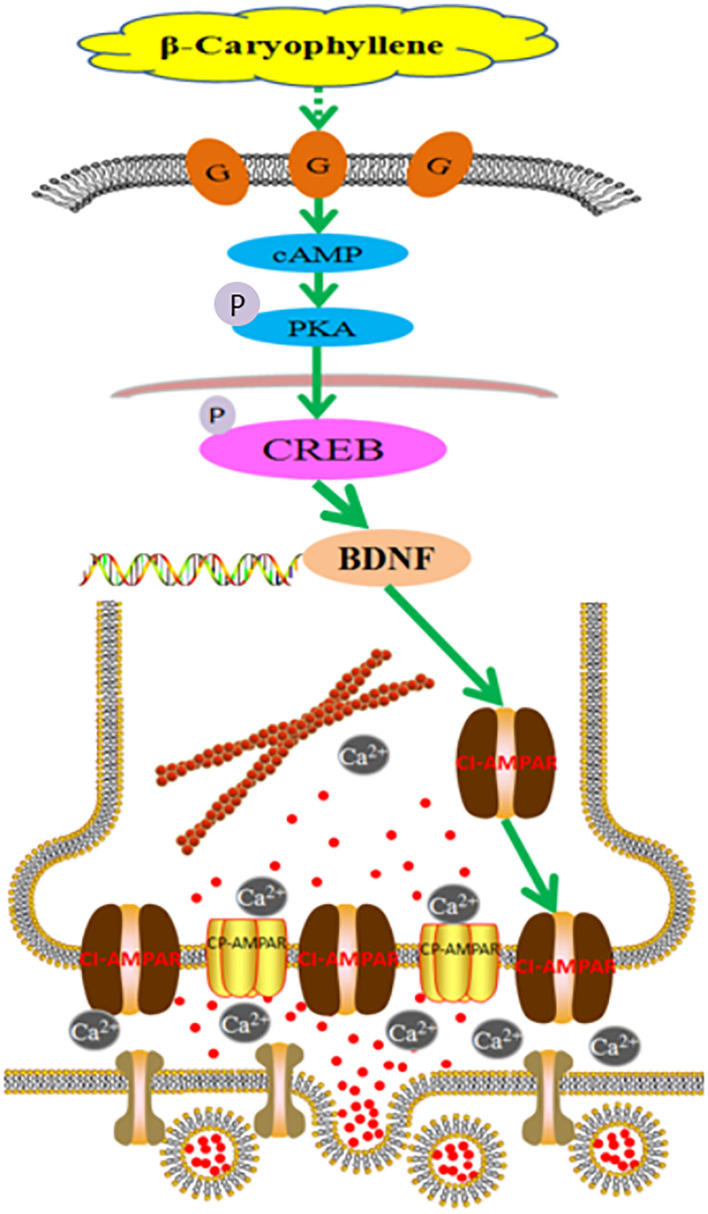
Molecular mechanism of β-caryophyllene in the treatment of post-acute ischemic stroke cognitive impairment via synaptic membrane surface recruitment of GluA2-containing Ca^2+^-impermeable AMPA receptor (CI-AMPARs). β-caryophyllene may activate the cAMP/PKA pathway, recruiting CI-AMPARs at the synaptic membrane to exert neuroprotective effects in acute ischemic stroke, which enhances the long-term potentiation effect and improves the learning memory abilities in post-acute ischemic stroke cognitive impairment.

To explore the molecular mechanism of BCP-mediated protection against the effects of acute ischemic stroke induced by MCAO, we added a group of mice treated with a PKA inhibitor, H-89. As shown in Figures 4A,B, H-89 significantly disrupted BCP-induced neuroprotection against acute ischemic stroke. Compared with the MCAO+BCP group, the neurological scores increased and the time spent on rota-rod test decreased. And cerebral infarction volume (Figures 4C,D) were increased and a large number of neuronal cells died in the hippocampal CA1 region in the MCAO+BCP+H-89 group (Figure 4E). No significant difference was observed between the H-89 inhibitor group and the MCAO group (^**^p < 0.01 vs. the sham group, ^*##*^*p* < 0.01 vs. the MCAO group, ^ΔΔ^*p* < 0.01 vs. the MCAO+BCP group). These results indicated that the cAMP/PKA pathway is involved in BCP-induced neuroprotection in acute ischemic stroke.

### The cAMP/PKA Pathway Is Involved in BCP-Induced Facilitation of CI-AMPAR Translocation to the Synaptic Membrane and Recruitment During Acute Ischemic Stroke

To determine whether the cAMP/PKA pathway is involved in the BCP-induced facilitation of CI-AMPAR translocation to the synaptic membrane and recruitment of these receptors in acute ischemic stroke, a western blot assay and confocal immunofluorescence analysis were performed. The western blot results showed that compared with the MCAO+BCP group, the MCAO+BCP+H-89 group exhibited significantly reduced the expression of the GluA2 AMPAR subunit (Figures 5A,B) on the surface of synaptic membranes, with a level similar to that of the MCAO group. Moreover, laser confocal immunofluorescence analysis also showed that compared with the MCAO+BCP group, the MCAO+BCP+H-89 group exhibited reduced colocalization of GluA2 and synaptophysin, with a level similar to that observed in the MCAO group (Figures 5C,D, ^**^p < 0.01 vs. the sham group, ^*##*^*p* < 0.01 vs. the MCAO group, ^ΔΔ^*p* < 0.01 vs. the MCAO+BCP group). Taken together, these results showed that H-89 can abolish BCP-mediated CI-AMPAR translocation to the synaptic membrane.

Additionally, we assessed the expression of cAMP by an ELISA and the expression of p-PKA, PKA, p-CREB, CREB, and BDNF by western blotting. As shown in Figures 6A–E (^**^p < 0.01 vs. the sham group, ^*##*^*p* < 0.01 vs. the MCAO group, ^ΔΔ^*p* < 0.01 vs. the MCAO+BCP group), compared with the MCAO+BCP group, the MCAO+BCP+H-89 group showed decreased expression of cAMP, p-PKA, p-CREB, and BDNF, which are not regulated by BCP, and the levels were similar to those in the MCAO group. Therefore, the cAMP/PKA pathway is involved in BCP-induced facilitation of CI-AMPAR translocation to the synaptic membrane and recruitment of these receptors during acute ischemic stroke.

### The cAMP/PKA Pathway Is Involved in BCP-Induced Inhibition of AMPAR-Mediated Miniature Excitatory Post Synaptic Currents (mEPSCs)

Changes in synaptic excitatory currents after MCAO and BCP treatment were analyzed using the patch clamp technique. Compared with the sham group, the amplitude and frequency of synaptic excitatory currents were higher in the MCAO group, as shown in Figures 7A–C. Compared with the MCAO group, the amplitude and frequency of synaptic excitatory currents were lower in the MCAO+BCP group. The results indicated that BCP can inhibit the AMPAR-mediated mEPSCs in the hippocampus neuron induced by acute ischemic stroke. Interestingly, we found that the cAMP/PKA signaling pathway inhibitor H-89 produced greater disruption the BCP-induced inhibition of AMPAR-mediated mEPSCs in the hippocampus neuron than in animals treated with BCP alone (^**^p < 0.01 vs. the sham group, ^*##*^*p* < 0.01 vs. the MCAO group, ^ΔΔ^*p* < 0.01 vs. the MCAO+BCP group). The results demonstrated that the cAMP/PKA pathway is involved in BCP-induced inhibition of AMPAR-mediated mEPSCs in the hippocampus. Because CI-AMPARs have neuroprotective effects because of their lower permeability for Ca^2+^ and because of the relative abundance of CI-AMPARs in AMPARs on the membrane surface, the changes in AMPAR-mediated synaptic excitatory currents depend on Ca^2+^ influx (37). Taken together, these results also further demonstrate that CI-AMPAR trafficking occurs in the acute ischemic stroke model, and BCP can facilitate CI-AMPAR translocation to the synaptic membrane and recruitment of these receptors via the cAMP/PKA pathway.

### Facilitation of CI-AMPAR Translocation to the Synaptic Membrane Surface, Which Depends on the cAMP/PKA Pathway, Is Required for BCP-Mediated Protection Against Post-acute Ischemic Stroke Cognitive Impairment

The effects of BCP on the spatial learning and memory abilities of post-acute ischemic stroke mice were examined using the MWM test. Without training, all mice reached the platform in the visible platform task on the 1st day. Before MCAO treatment, no significant difference was observed in the spatial learning abilities of different groups of mice, and their spatial learning abilities gradually increased with increasing training time (Figure 8A). However, at 7 days after MCAO, the spatial learning ability of the MCAO group was significantly impaired compared with the sham group (Figure 8B), and the escape time was significantly increased. Compared with the MCAO group, the BCP group showed significantly shorter the escape time. Moreover, in the MCAO+BCP+H-89 group, we found that H-89 could counteract the shorter escape time induced by BCP treatment, resulting in no significant difference between the H-89-treated group and the MCAO group (^**^p < 0.01 vs. the sham group, ^*##*^*p* < 0.01 vs. the MCAO group, ^ΔΔ^*p* < 0.01 vs. the MCAO+BCP group). Next, the platform was removed, and the swimming track and the number of times that the platform location was crossed were recorded. MCAO group mice exhibited fewer platform crossings and spent less time in the target quadrant than the sham group. However, BCP treatment significantly increased the platform crossings and time spent in the target quadrant. Moreover, H-89 could reverse the effects induced by BCP treatment, as shown by the MCAO+BCP+H-89 group (Figures 8C–E, ^**^p < 0.01 vs. the sham group, ^*##*^*p* < 0.01 vs. the MCAO group, ^ΔΔ^*p* < 0.01 vs. the MCAO+BCP group). The results of the H-89-treated group were not significantly different from those of the MCAO group. These results demonstrated that translocation of CI-AMPARs to the synaptic membrane surface depends on the cAMP/PKA pathway, and this translocation is required for BCP-mediated protection against post-acute ischemic stroke cognitive impairment.

## Discussion

Cognitive impairment is a frequent condition after acute ischemic stroke (38), and its prevalence ranges from 6 to 32% (1). The MCAO model is often employed to simulate acute ischemic stroke. Many mechanisms had been shown to be involved in the pathophysiology of post-acute ischemic stroke cognitive impairment (39). Currently, changes in synaptic function are the main therapeutic targets for cognitive dysfunction (40). This synaptic plasticity is the structural basis of synaptic function, which is expressed as LTP and LTD. AMPARs are involved in LTP and LTD, which mainly occur in the CNS and are responsible for the transmission of CNS information (41).

AMPARs are ion channels that bind excitatory amino acids, mainly including glutamate. In acute ischemic stroke, glutamate is released into the intercellular space, resulting in a 100-fold increase in the concentration of glutamate in this space. A high concentration of the glutamate leads to inflow of Ca^2+^, causing lethal injury in nerve cells (42, 43). Under basal conditions in wild-type mice, GluA1/GluA2- and GluA2/GluA3-containing AMPARs mediate ~80 and 20%, respectively, of postsynaptic responses in the hippocampus. These AMPARs are Ca^2+^-impermeable (CI-AMPARs) because of the presence of GluA2 and show little if any inward rectification (44). Thus, the GluA2 subunit dictates the critical biophysical properties of AMPARs, strongly influences AMPAR assembly and trafficking, and plays pivotal roles in a number of forms of long-term synaptic plasticity. An alteration in the level of GluA2 expression produces a change in the Ca^2+^ permeability and in the waveform of the synaptic current, leading to a qualitative change in synaptic transmission. Incorporation of GluA2 subunits to plasma membrane played a key role in transient forebrain ischemia by regulating Ca^2+^ permeability (45).CI-AMPARs block the inflow of Ca^2+^ and alleviate this injury (46). Down-regulation CI-AMPARs on the cell surface increases Ca^2+^ permeability and may be a major mechanism in cellular models of acute and repeated hypoxic-ischemic injury (47). Hippocampal neurons have been reported to exhibit internalization of CI-AMPARs, and translocation of CP-AMPARs to the synaptic membrane is facilitated in these neurons; therefore, these mechanisms may be relevant to ischemia-induced neuronal injury and death (48). In this paper we focused the study of post-acute ischemic stroke cognitive impairment and the role of BCP on it. So, we assessed the expression of GluA2 at first in this study based on its important role in determining Ca^2+^ flux, and Ca^2+^ also has a key role in acute ischemic stroke. In this study, by examining the TTC and H&E staining results and neurological deficits, rotarod test we found that BCP treatment decreases the ischemic area in the brain in a mouse model of acute ischemic stroke. In addition, a decrease in the expression of CI-AMPARs on the synaptic membrane was observed in the acute ischemic stroke group. However, BCP could increase the expression of CI-AMPARs on the synaptic membrane surface. These results demonstrated that BCP is associated with CI-AMPAR translocation to the synaptic membrane surface and recruitment of these receptors, which is essential for their role in neuroprotection.

Moreover, CI-AMPARs move rapidly between the postsynaptic membrane surface and the intracellular environment via regulated receptor endocytosis and exocytosis, which is a mechanism that is critical for many forms of synaptic plasticity including LTP and LTD. Our previous studies showed that BCP can alleviate cognitive impairment and memory deficits caused by chronic cerebral ischemia in SD (Sprague-Dawley)and AD mice by stimulating CB2Rs (24, 25). Incorporation of the GluA2 subunits into an AMPA receptor alters a number of key biophysical properties, including Ca^2+^ permeability and the waveform of the synaptic current. These changes alter the ability of synaptic currents to evoke an action potential and therefore have a profound effect on the computational capability of individual neurons and thus the output of neuronal circuits (49). Of the four AMPA receptor subunits, incorporation of the GluA2 subunit reduces the Ca^2+^ permeability and channel conductance and prolongs the decay kinetics of a synaptic current (50). It also was reported that a rapid synaptic insertion of CP-AMPARs and a decrease of CI-AMPARs effect enhanced AMPAR-mediated excitatory postsynaptic current (EPSC_*AMPA*_) (51). In this study, analysis of changes in synaptic excitatory currents after MCAO and BCP treatment using patch clamp recordings showed that BCP can inhibit the AMPAR-mediated mEPSCs in the hippocampus neuron induced by acute ischemic stroke. In addition, the MWM test results also indicated that BCP treatment markedly improved the learning and memory abilities of acute ischemic stroke mice. These results suggested that BCP has protective effects against acute ischemic stroke.

The cAMP/PKA pathway is important for learning and memory abilities (26). Our previous work presented that CB2R activation lead to PI3K/AKT and PPARγ pathway. And there are some cross-talk or upstream/downstream relationship between the PKA and PI3K/AKT and PPARγ pathway (52, 53). In addition, studies have demonstrated that CI-AMPARs undergo constitutive insertion, which is accelerated by PKA signaling, and the increase in GluA2 insertion after PKA activation must reflect insertion of CI-AMPARs (30). PKA phosphorylation also mediates the increase in CI-AMPARs in hippocampal neurons (31). CREB is involved in the transformation from short-term memory to long-term memory (32). BDNF is a key factor that regulates synaptic plasticity in hippocampal neurons and improves cognitive and affective disorders. BDNF can transport AMPARs to the synaptic site to exert LTP, thereby improving learning and memory abilities (54). CI-AMPARs undergo constitutive insertion that is accelerated by PKA signaling, extending previous results obtained by measuring GluA1 insertion (30). In recent years, many studies have shown that activation of the CB2R is associated with changes in the BDNF pathway. Activation of the CB2R was reported to activate the downstream cAMP/PKA pathway and reduce cerebral ischemic injury (28). CB2R agonists have also been shown to improve subarachnoid hemorrhage by activating CREB to inhibit apoptosis (55). The key molecules of the cAMP/CREB/BDNF pathway play important role in synaptic plasticity. We focused the study of cognitive impairment in this paper. Thus, we analyzed if the key molecules of the cAMP/CREB/BDNF pathway changed in MCAO mice model or/and treating with BCP, a full agonist of the CB2R.

In our study, we observed the effects of BCP on the cAMP/PKA pathway in an acute ischemic stroke mouse model. A notable decrease in expression of the cAMP, p-PKA, p-CREB, and BDNF was observed in the acute ischemic stroke mouse model (MCAO group) compared with the sham group. However, BCP obviously promoted expression of these molecules in the MCAO+BCP group, indicating that BCP can activate the cAMP/PKA pathway in an acute ischemic stroke mouse model. To further examine this phenomenon, a selective PKA inhibitor (H-89) was used. Interestingly, we found that H-89 significantly disrupted BCP-induced neuroprotection against acute ischemic stroke. Additionally, consistent with this neuroprotective effect, H-89 decreased the BCP-mediated translocation of CI-AMPARs to the synaptic membrane surface. Furthermore, H-89 significantly disrupted the BCP-induced decrease in the amplitude and frequency of synaptic excitatory currents mediated by AMPARs. The MWM test results also indicated that H-89 disrupted the effect of BCP treatment on improving the learning and memory abilities of post-acute ischemic stroke mice. These data indicate that PKA-dependent synaptic membrane surface recruitment of CI-AMPARs is crucial for the neuroprotective effect of BCP against acute ischemic stroke as well as for protection against post-acute ischemic stroke cognitive impairment.

In summary, the above results indicate that BCP may activate the cAMP/PKA pathway (Figure 9), causing CI-AMPARs at the synaptic membrane surface to exert neuroprotective effects in acute ischemic stroke, thus regulating LTP and LTD and improving the learning and memory abilities in individuals with post-acute ischemic stroke cognitive impairment. Synaptic membrane surface recruitment of CI-AMPARs serves as putative therapeutic targets for acute ischemic stroke and protection against post-acute ischemic stroke cognitive impairment, and the results of this study provide new insights for the application of BCP as a neuroprotective agent.

## Data Availability Statement

The original contributions presented in the study are included in the article/Supplementary Material, further inquiries can be directed to the corresponding author.

## Ethics Statement

The animal study was reviewed and approved by Experimental Ethics Committee of Chongqing Medical University.

## Author Contributions

ZD designed the study. SC performed most of the research study and wrote the manuscript. YW performed the animal experiments. XW analyzed the data. MH performed the western blotting experiments. LZ performed the patch clamp experiments. All authors contributed to the article and approved the submitted version.

## Conflict of Interest

The authors declare that the research was conducted in the absence of any commercial or financial relationships that could be construed as a potential conflict of interest.
