# Supplementary figures and images for: PKA-Dependent Membrane Surface Recruitment of CI-AMPARs Is Crucial for BCP-Mediated Protection Against Post-acute Ischemic Stroke Cognitive Impairment
Source: Front Neurol. 2020 Dec 16;11:566067. doi: 10.3389/fneur.2020.566067 (PMC7772322; doi:10.3389/fneur.2020.566067)

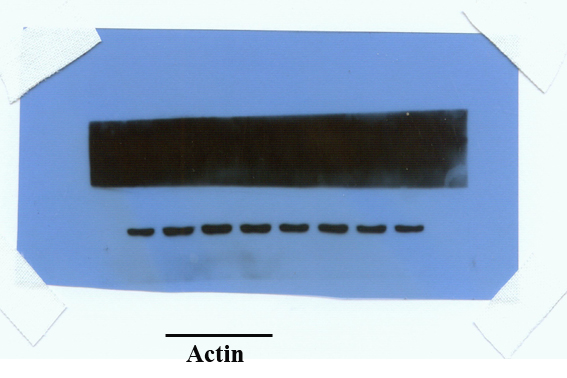

Supplement: Supplementary file 1 [file Data_Sheet_1.ZIP › 6.02-Blot raw data/fig2A/fig2A-actin.jpg]

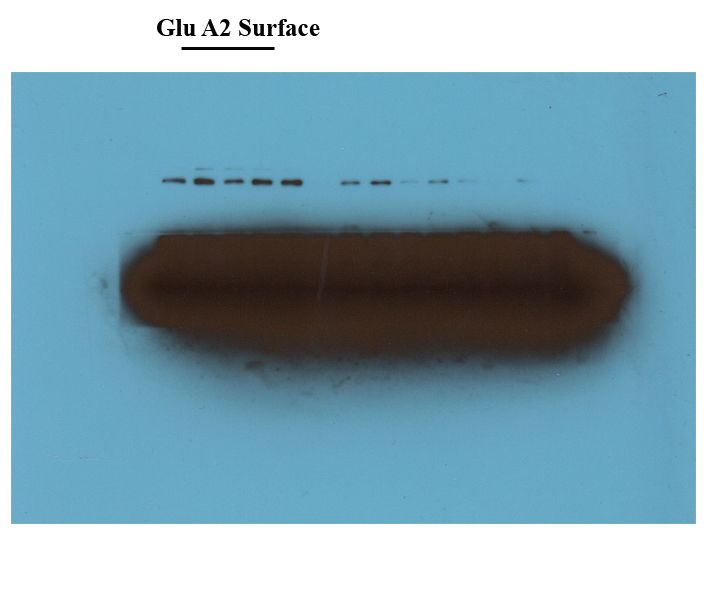

Supplement: Supplementary file 1 [file Data_Sheet_1.ZIP › 6.02-Blot raw data/fig2A/fig2A-GluA2 Surface.jpg]

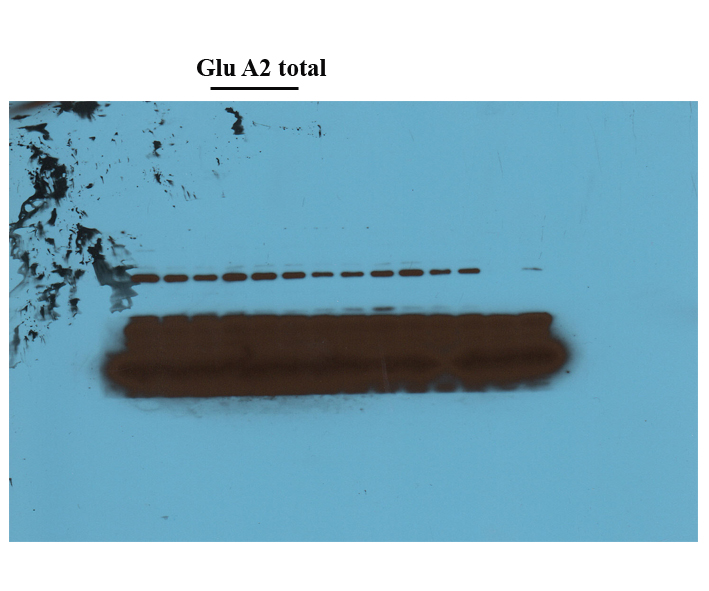

Supplement: Supplementary file 1 [file Data_Sheet_1.ZIP › 6.02-Blot raw data/fig2A/fig2A-GluA2 total.jpg]

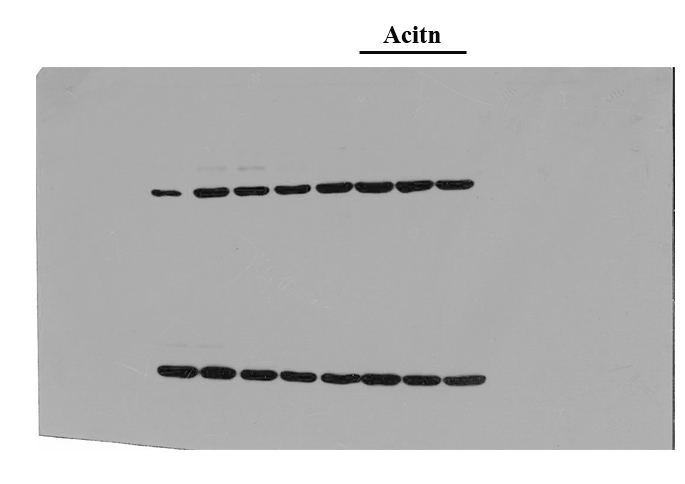

Supplement: Supplementary file 1 [file Data_Sheet_1.ZIP › 6.02-Blot raw data/fig3B/fig3B-Actin.jpg]

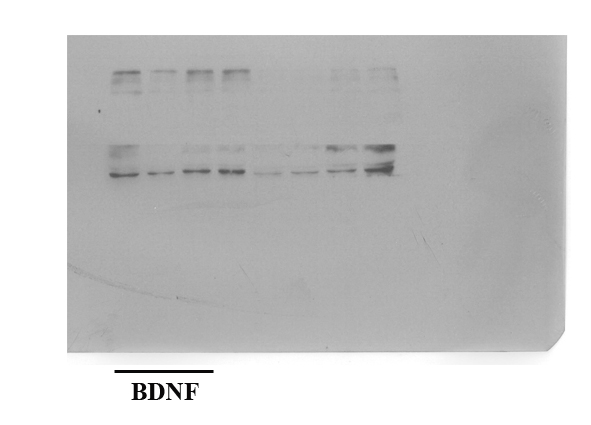

Supplement: Supplementary file 1 [file Data_Sheet_1.ZIP › 6.02-Blot raw data/fig3B/fig3B-BDNF.jpg]

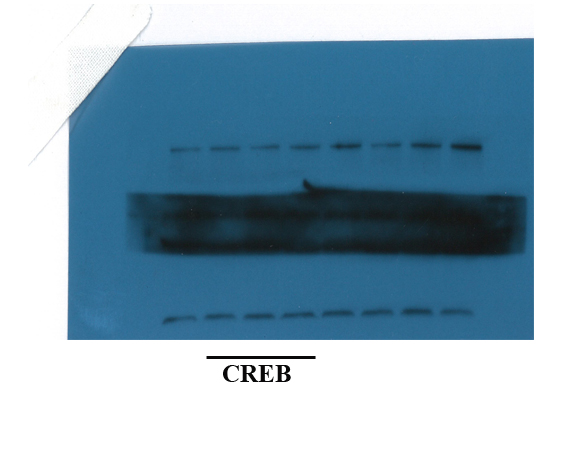

Supplement: Supplementary file 1 [file Data_Sheet_1.ZIP › 6.02-Blot raw data/fig3B/fig3B-CREB.jpg]

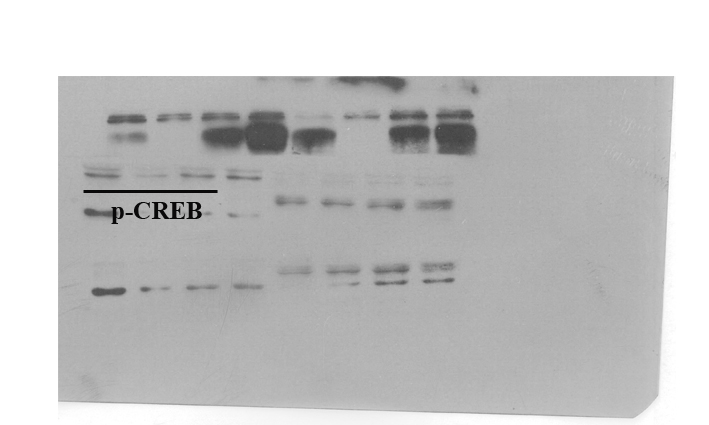

Supplement: Supplementary file 1 [file Data_Sheet_1.ZIP › 6.02-Blot raw data/fig3B/fig3B-p-CREB.jpg]

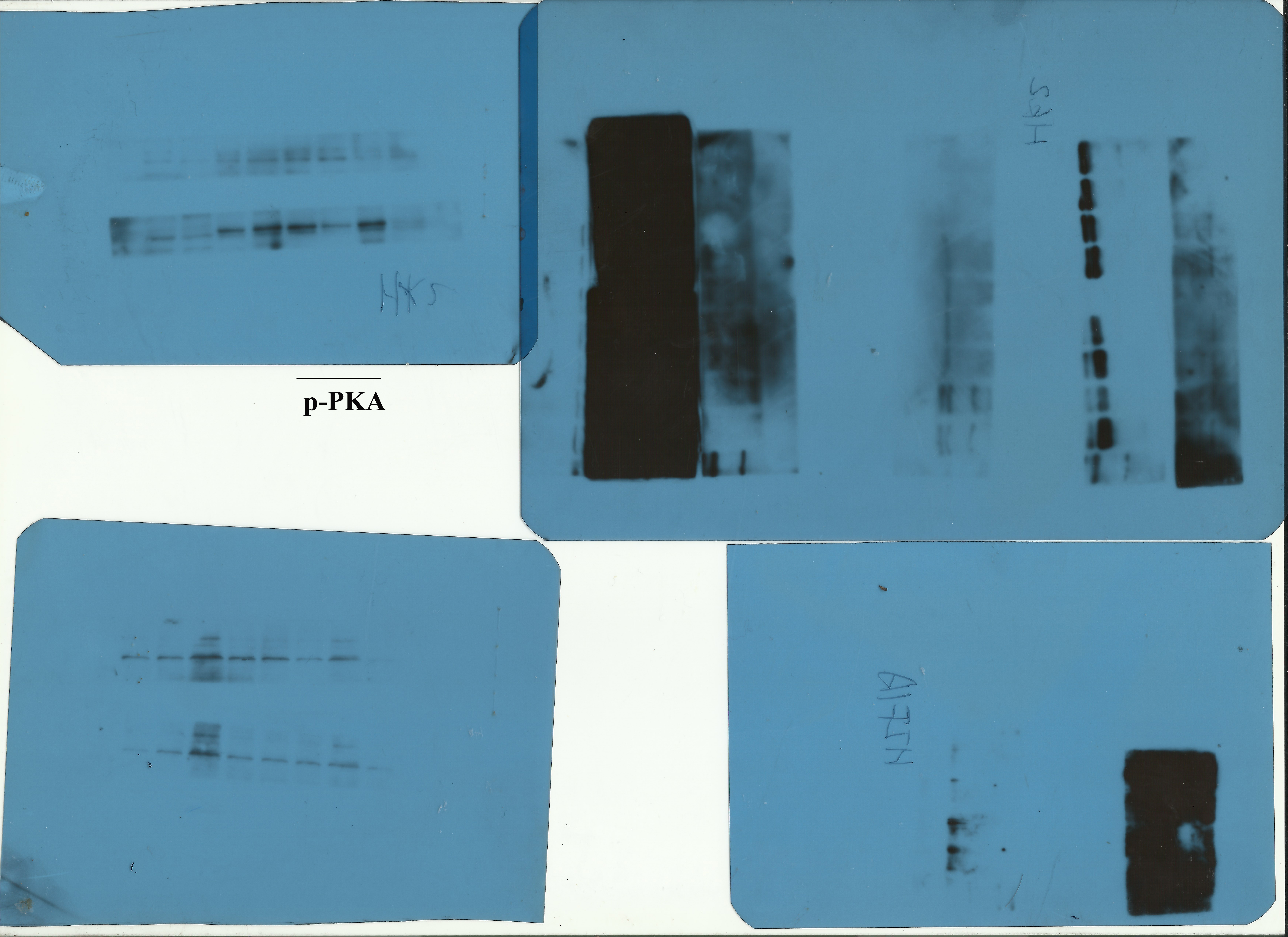

Supplement: Supplementary file 1 [file Data_Sheet_1.ZIP › 6.02-Blot raw data/fig3B/fig3B-P-PKA.jpg]

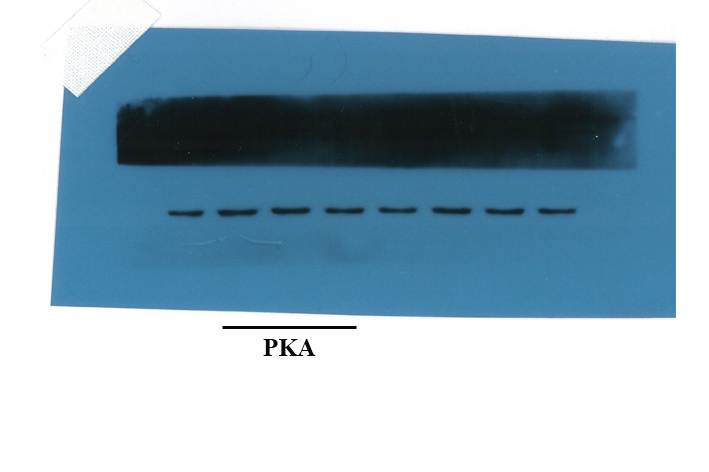

Supplement: Supplementary file 1 [file Data_Sheet_1.ZIP › 6.02-Blot raw data/fig3B/fig3B-PKA.jpg]

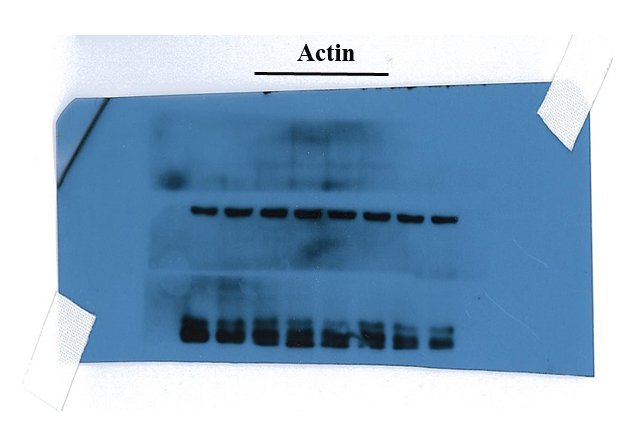

Supplement: Supplementary file 1 [file Data_Sheet_1.ZIP › 6.02-Blot raw data/fig5A/fig5A-Actin.jpg]

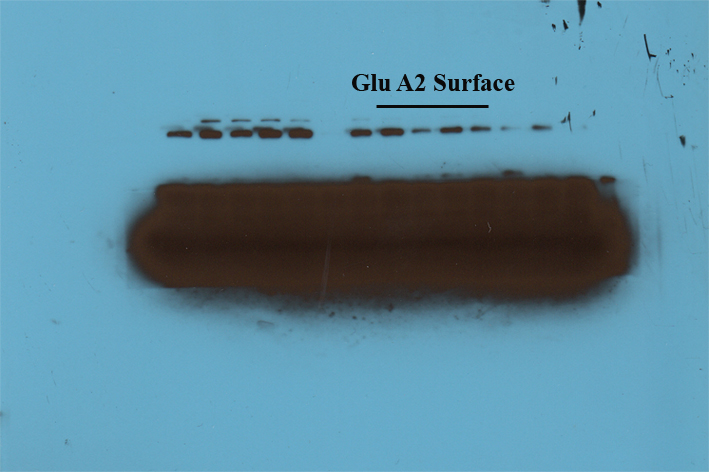

Supplement: Supplementary file 1 [file Data_Sheet_1.ZIP › 6.02-Blot raw data/fig5A/fig5A-GluA2 Surface.jpg]

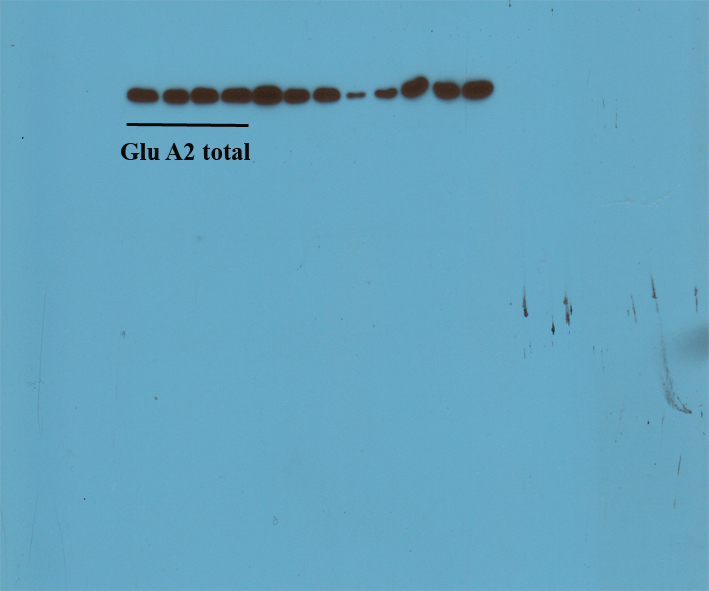

Supplement: Supplementary file 1 [file Data_Sheet_1.ZIP › 6.02-Blot raw data/fig5A/fig5A-GluA2 total.jpg]

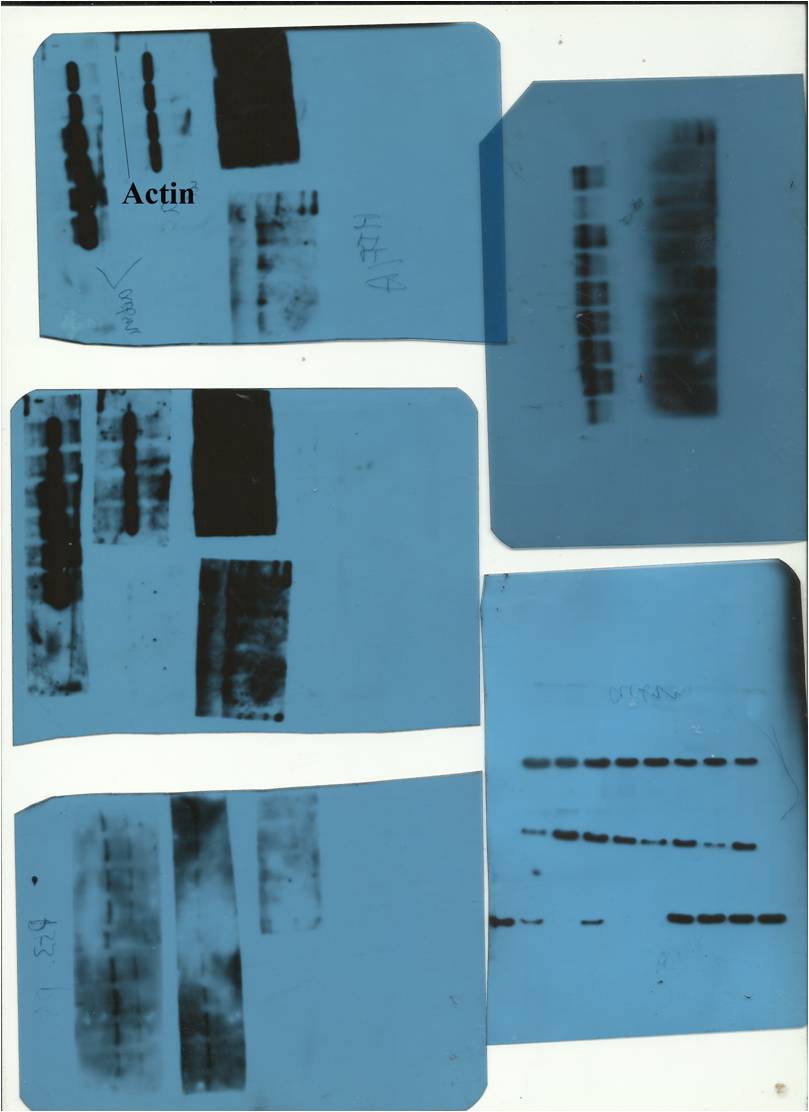

Supplement: Supplementary file 1 [file Data_Sheet_1.ZIP › 6.02-Blot raw data/fig6B/fig6B-Actin.jpg]

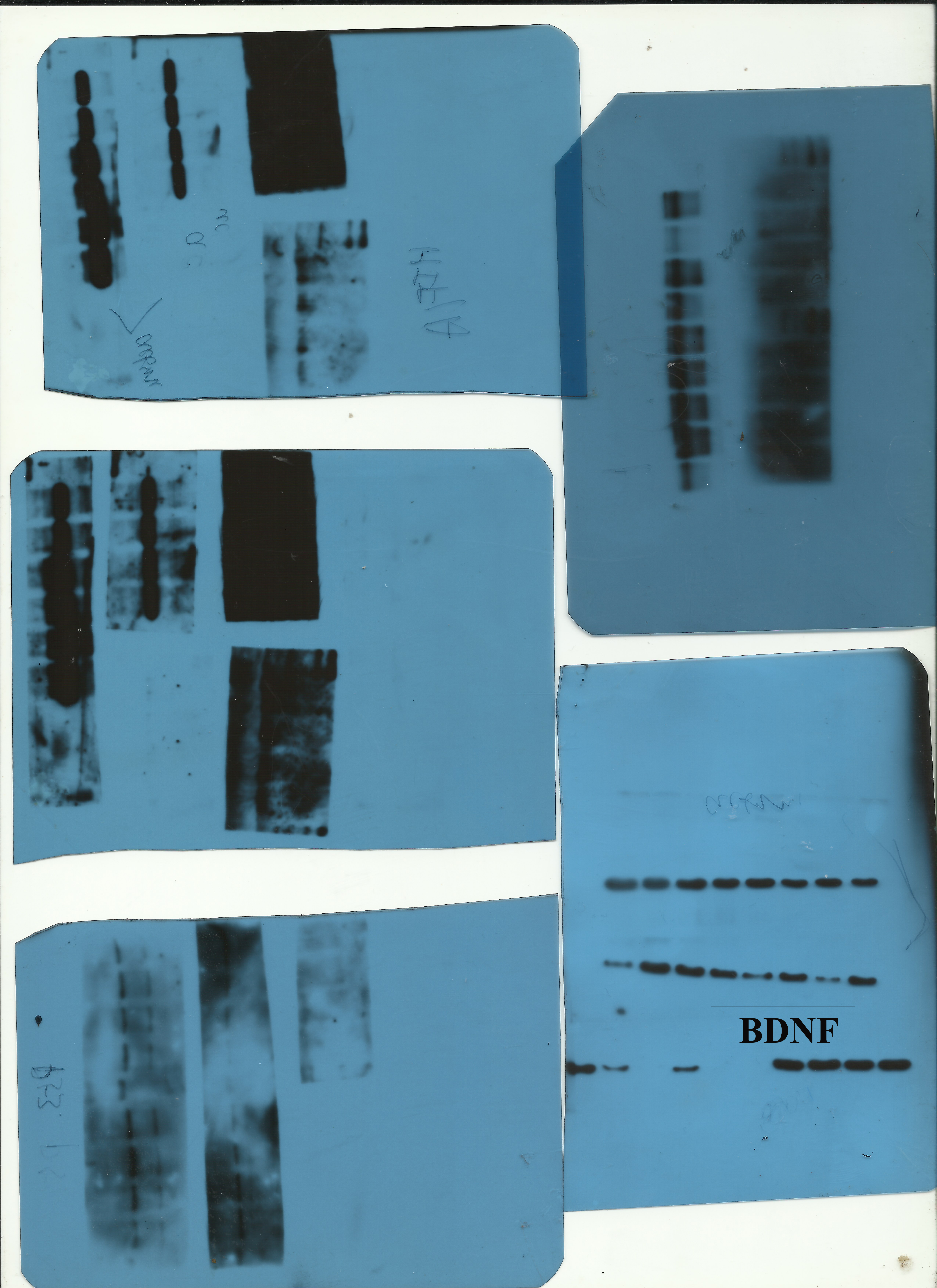

Supplement: Supplementary file 1 [file Data_Sheet_1.ZIP › 6.02-Blot raw data/fig6B/fig6B-BDNF.jpg]

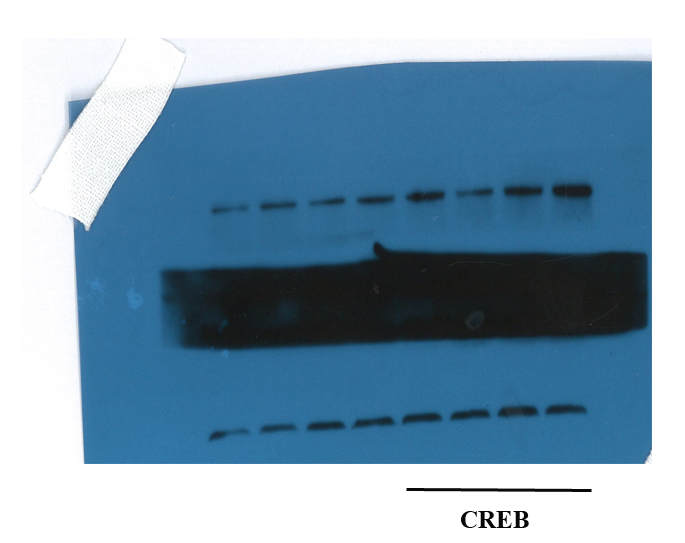

Supplement: Supplementary file 1 [file Data_Sheet_1.ZIP › 6.02-Blot raw data/fig6B/fig6B-CREB.jpg]

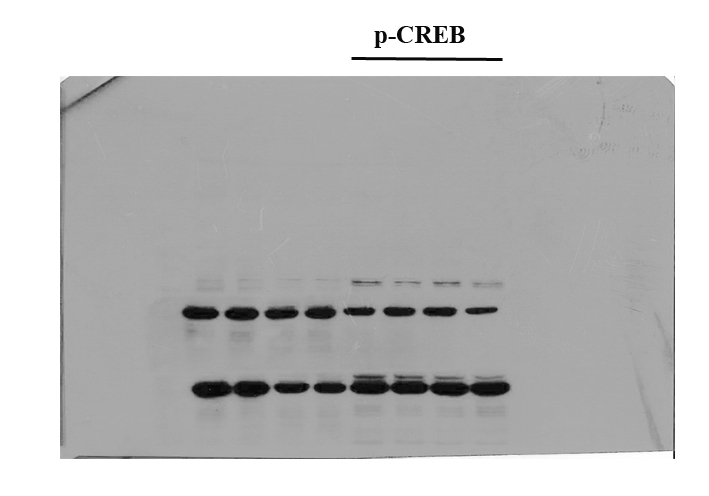

Supplement: Supplementary file 1 [file Data_Sheet_1.ZIP › 6.02-Blot raw data/fig6B/fig6B-p-CREB.jpg]

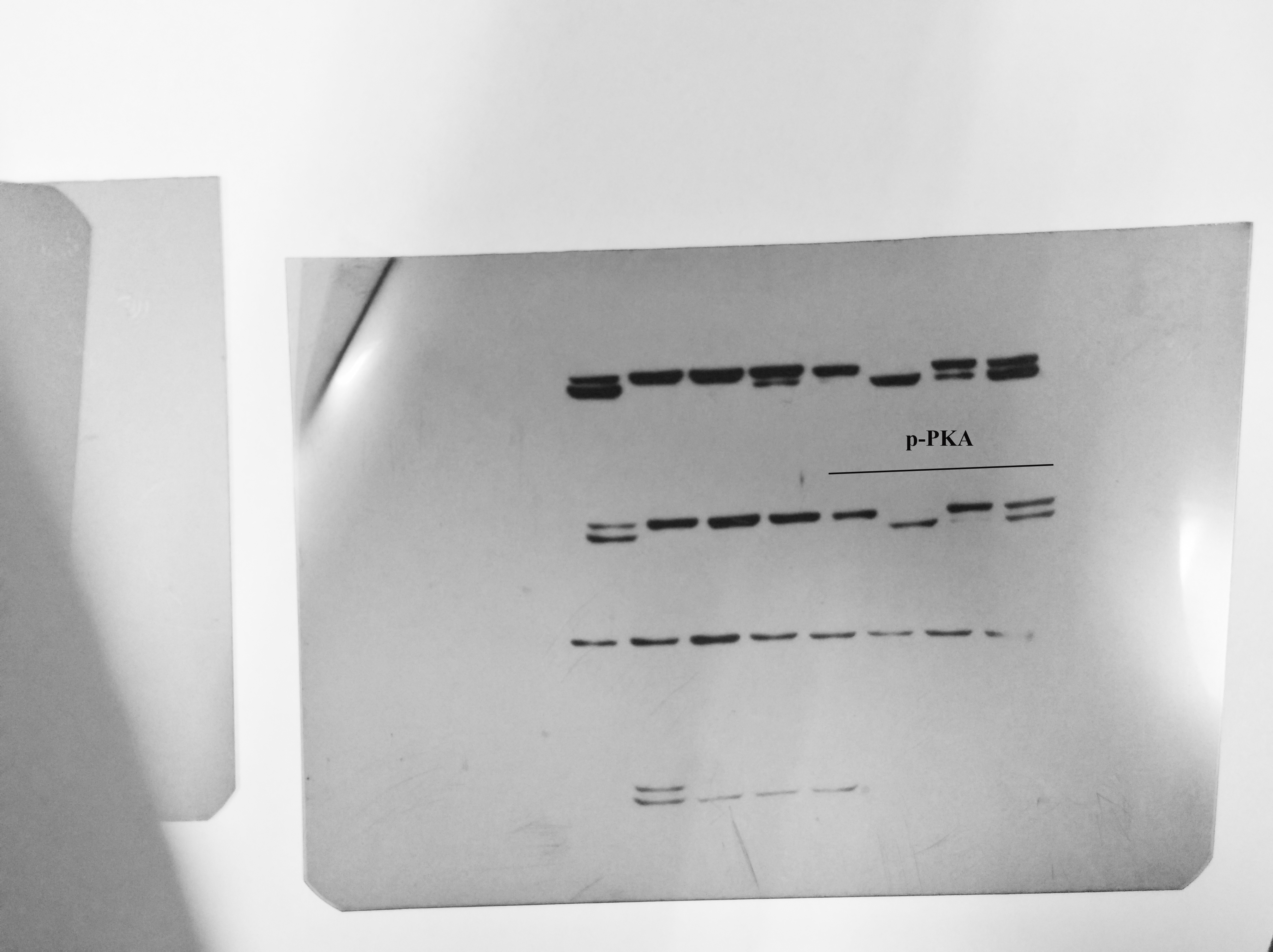

Supplement: Supplementary file 1 [file Data_Sheet_1.ZIP › 6.02-Blot raw data/fig6B/fig6B-p-PKA.jpg]

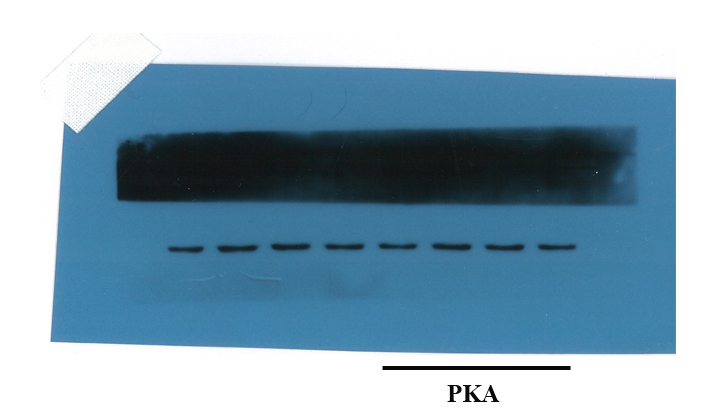

Supplement: Supplementary file 1 [file Data_Sheet_1.ZIP › 6.02-Blot raw data/fig6B/fig6B-PKA.jpg]
